# Bilateral inverted papilloma: Case report and literature review

**DOI:** 10.1016/j.radcr.2024.11.053

**Published:** 2024-12-21

**Authors:** Mohammed Amine Rabhi, Achraf Amine Sbai, Drissia Benfadil, Azzedine Lachkar, Adil Abdenbi Tsen, Fahd Elayoubi

**Affiliations:** a Faculty of Medicine and Pharmacy, Mohammed First University, Oujda, Morocco; b Department of Ear Nose and Throat, Mohammed VI University Hospital Mohammed First University, Oujda, Morocco; c Mohammed First University, Faculty of Medicine and Pharmacy, LAMCESM, Oujda, Morocco; d Departement of Maxillofacial Surgery, Mohammed VI University Hospital Mohammed First University, Oujda, Morocco

**Keywords:** Inverted papilloma, Nasal obstruction, Benign nasal tumor, Paranasal sinus

## Abstract

Inverted papilloma is a rare, benign epithelial tumor of the nasal and sinus cavities with an unclear etiology. It usually presents as unilateral nasal obstruction. Diagnosis is histological, and treatment is primarily surgical. We describe a 72-year-old patient with persistent bilateral nasal obstruction. Examination revealed a greyish-pink, multi-lobed mass in both nasal cavities pressing on the hard palate. Imaging (CT and MRI) showed a cerebriform mass extending through the nasal cavities and into the nasopharynx, causing bone destruction without intracranial or intra-orbital spread. Histopathology confirmed a bilateral inverted papilloma without malignancy. Inverted papilloma comprises less than 4% of primary nasal tumors. It is noted for high local aggressiveness, frequent recurrences, and potential association with squamous cell carcinoma. Imaging (CT and MRI) aids in staging and selecting the surgical approach—endoscopic, external, or combined. Close surveillance via endoscopy and imaging is crucial. Although benign, inverted papilloma is highly invasive. Accurate diagnosis and complete surgical removal are essential to minimize recurrence risk.

## Introduction

The enormous variation in the clinical, radiological, and notably anatomopathological presentations of tumors of the nasal cavity and paranasal sinuses is what makes them so distinctive [1]. The inverted papilloma is a rare benign tumor that develops from the Schneiderian mucosa of the nasal cavity and most commonly affects individuals in their fifth decade [2]. It was initially identified in 1854 by WARD et al. It is distinct from other nasal-sinusal tumors mainly for 3 reasons: relative local aggressiveness, a high recurrence rate, whether early or late, and a probable association with carcinoma [3]. Although the precise etiology is still unknown, over 40% of cases have been linked to human papillomavirus [4]. The most frequent symptom is chronic unilateral nasal obstruction. The other symptoms will depend on the local extension of the lesion. The diagnosis is based on the anamnesis, a complete ENT examination, a CT-MRI scan to establish the local extension of the tumor, and histological confirmation [2]. The surgical technique may be endoscopic endonasal or an external approach, depending on the extent and characteristics of the tumor.

This report discusses the case of a patient with an extremely aggressive bilateral inverted papilloma.

## Case presentation

**Fig. 1:**
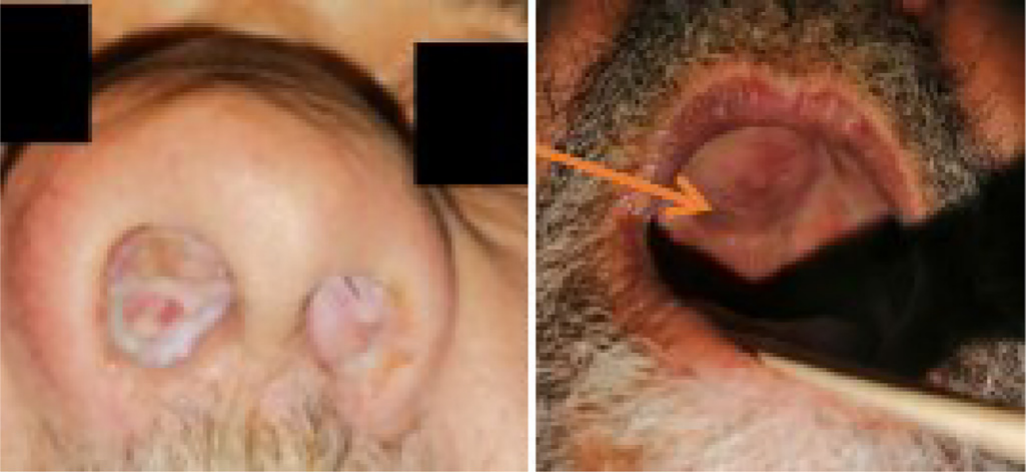
Clinical image showing a pinkish-gray, multilobed mass deforming the nasal pyramid and exerting a mass effect on the hard palate.

A 72-year-old man, a chronic smoker, has been suffering from chronic nasal obstruction for the past 15 years. It started out unilateral on the right side, and spread to both nasal cavities after a year, accompanied by mucopurulent rhinorrhea, hyposmia and bilateral hypoacusis. However, the patient did not suffer from epistaxis, headaches, or visual impairment. On the basis of clinical examination, the patient presents bilateral mucopurulent rhinorrhea, hypertelorism and deviation of the nasal wings. The flexible endoscope cannot pass through both nostrils, which are completely filled with a fleshy, greyish-pink, poly-lobed mass that is painless and nonbleeding on contact. On endobuccal examination, the hard palate shows a firm, painless swelling that is lateralized to the right, seromucous otitis on otoscopy, and no inflammatory symptoms opposite (Fig. 1).

**Fig. 2:**
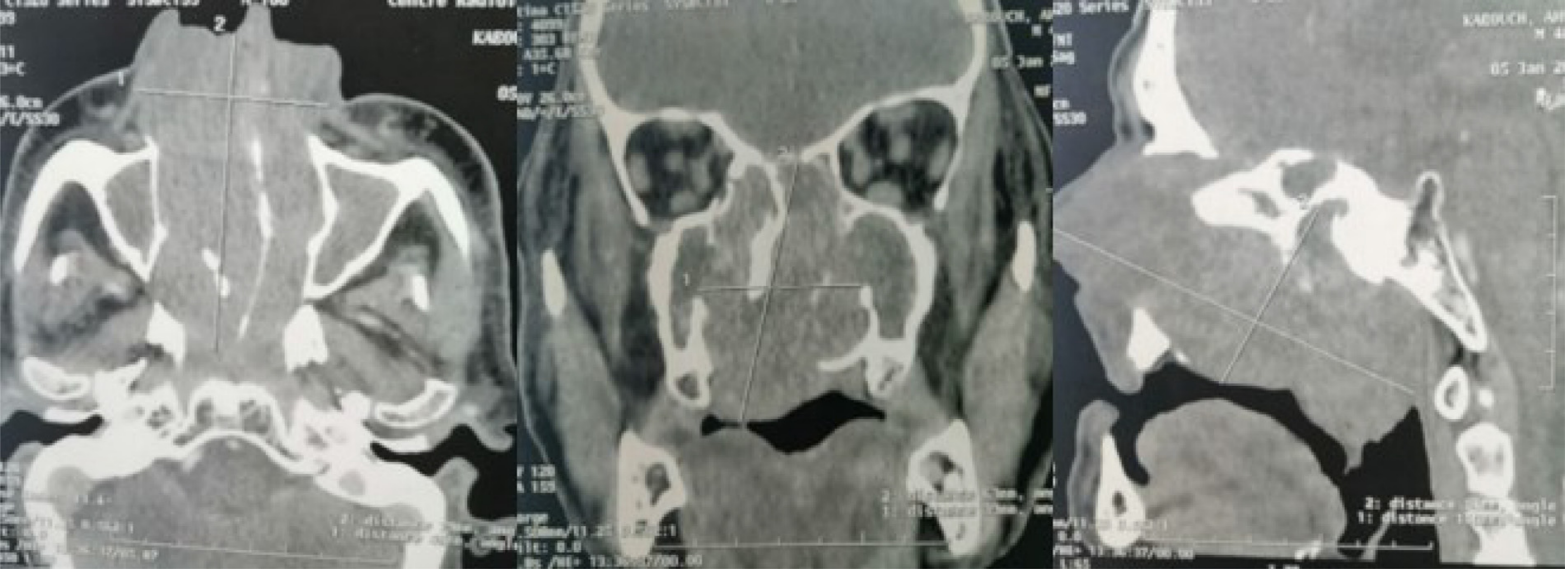
Axial, coronal, and sagittal sections of a nasal scan displaying the invasion of the 2 nasal cavities by an expansive process extending laterally to the 2 maxillary sinus by lysing their walls, inferiorly to the nasal floor by breaking the bone palate, superiorly the mass comes into the riddled blade of the ethmoid without breaking it, without intracranial or infraorbital extension.

Nasal and paranasal sinuses CT-MRI pair was advised and revealed a tissue ceribriform mass, taking the totality of the 2 nasal cavities extended to the cavum, without intracranial or intra-orbital extension, responsible for a bone lysis of the nasal septum, ethmoidal cells, internal walls of the orbits and of the 2 maxillary sinuses, pterygoid blades and hard palate. Under local anesthetic, a nasal biopsy was taken and sent for histopathological study, showing an inverted papilloma with no sign of malignancy (Fig. 2).

The course of treatment included the excision of the tumor using a rigorous endoscopic technique using 0° and 30° optics. After the intranasal component was expelled in 2 blocks, a bilateral maxillectomy was performed to allow for the expulsion and control of the intra sinus parts. The 2 sphenoids sinus and the anterior and posterior ethmoids were further explored. A Dessi nasal Bipolar Forceps helped to facilitate dissection, lessen bleeding, and maintain hemostasis at the end of the procedure. A bilateral inverted papilloma was identified on histological examination without any evidence of cell atypia.

The postoperative evolution was acceptable, and the patient was allowed to leave the hospital on the seventh postoperative day under nasal lavage. A nasofibroscopy every 2 months for the first 6 months, followed by every 6 months, has objectively proven that the 2 nasal cavities have healed well. After a year, a CT of the sinuses were ordered. The results were normal, with no local recurrence.

## Discussion

Inverted papilloma, also known as schneider cell papilloma, is a benign epithelial tumor that accounts for 0.5%-4% of all primary nasal tumors, and is unilateral in 91%-99% of cases [4]. It develops from the Schneiderian membrane that lines the lateral wall of the nasal cavity through a mechanism that causes the superficial epithelium to invert into the underlying stroma, hence the name « inverted papilloma » [5]. the prevalence rate is highest in the fifth and sixth decades, with a significant male predominance.

Despite being a benign tumor, it is known for its destructive capacity and aggressive growth, which can cause bone damage in surrounding tissues [6]. Its probability of recurrence after excision, with rates ranging from 0% [7] to 78% [8] especially in cases of involvement of the frontonasal duct, the supraorbital ethmoids, the lacrimal fossa, and the infraorbital recess of the maxillary sinus [9]. Moreover, its ability to progress malignantly and be linked to squamous cell carcinoma, which was found to be associated with an incidence of 21% in the Myers and al [5] trial and 53% in the Yamaguchi and al study [10].

The etiology of IP is currently unknown [11]. One possible explanation is that Schneider's membranes have undergone numerous modifications that increase their susceptibility to neoplastic differentiation [12]. Other probable factors have been mentioned, including smoking, allergies, occupational exposures, infections or inflammatory rhinosinusitis [11]. Recurrence and the possibility of carcinomatous growth have long indicated a viral origin, involving human papillomavirus (HPV) subtypes 6, 11, 16 and 18, as well as Epstein-Barr virus (EBV) [13].

IP is generally diagnosed at a later stage, 1 to 4 years after the onset of sinonasal symptoms [2]. Unilateral nasal obstruction is the most frequent presenting symptom, its percentage in the literature ranges from 64% [14] to 81% [15]. Additional clinical findings include rhinorrhea (17%), epistaxis (6%), anosmia (4%), headache and frontal pain [6]. Within the nasal cavity, the tumor can spread to the exterior in 7% of cases, the nasopharynx in 3% of cases, and the pterygopalatine and cerebral fossa in fewer than 2% of cases [3]. Clinical evaluation by endoscopic exploration of the nasal cavity reveals a lobulated, exophytic tumor, ranging in consistency from firm to friable, and in color from pink to gray [4].

Pathologic examination is crucial to diagnosis and establishing the treatment strategy by seeking out a potential association with squamous cell carcinoma. Based on their morphology, benign papillomas can be classified into 3 types, the fungiform or exophytic, which originates from the anterior septum and resembles a common wart in macroscopic terms; the columnar or oncocytic, which originates from the lateral nasal wall and the middle meatus; and the inverted, which arises from the Schneiderian membrane [16].

The 2 primary goals of radiological examination are to pinpoint the exact location of the tumor site and provide specific information about its full extension. Despite not being pathognomonic, the inverted papilloma frequently exhibits a characteristic radiological appearance [17]. The most common finding on CT is an isodense homogenous lesion that often extends from the middle meatus via an expanded maxillary ostium and into the neighboring maxillary sinus [5]. CT scans may additionally detect bone destruction, which is a very typical feature of this tumor, and look for areas of microcalcifications, presents in 20% of cases [18]. MRI in now systematic on IP's assessment, it provides tumor boundaries with more accuracy, determinates the intracranial extension, distinguishes the lesion from the inflammatory tissue, for that reason, it is the preferred exam for postoperative follow-up [19]. The IP shows an aspect of cerebriform circumvolutions, seen on both T2 and contrast-enhanced T1 weighted images [20].

Numerous classifications of inverted papillomas have been reported in the literature, such as those made by Kamel (2005), Cannady (2007), and Dragonetti (2011). Because of its simplicity and reliability, the KROUSE classification published in 2000 is the most often used method. It divides the tumor into 4 categories based on the tumor's invasion of the paranasal sinuses and potential for malignant transformation [21].

There is general consensus that inverted papillomas are best managed and controlled by complete surgical excision [22]. Previously, we used an external strategy, typically paralateronasal with a corresponding medial maxillectomy. Treatments became less intrusive with the advent of the endoscopic approach, which is now considered the gold standard by many authors, nevertheless with a higher recurrence rate [23]. Stankwigcz and al [24], proposed endoscopic surgery only in the cases of unilateral disease without malignancy characteristics, confined to the middle meatus and middle turbinate. Otherwise, an external or combined external/endoscopic approach should be considered whenever total endoscopic control of the IP appears impossible [25] (Table 1). therapeutic effectiveness depends on complete exposure of the tumor insertion point, allowing total resection with ideally removing bone and the adjacent periosteum at disease site, which can be perfectly done by burring the bone with a diamond drill [26].

**Table 1:** Surgical approach according to tumor extension.

Involvement	Suggested surgical approach
Septum Lateral wall of nasal cavity Anterior or posterior ethmoid Sphenoethmoid and sphenoid spaces Maxillary sinus (medial, superior or posterior wall) Frontal space and frontal sinus (limited medial involvement)	Endonasal endoscopic
Lateral wall of frontal sinus	Endonasal endoscopic + frontal osteopastic flap (e.g: bicoronal approach)
Maxillary sinus (anterior, inferior or lateral wall)	Endonasal endoscopic + caldwell-luc approach
Extrasinus extension Associated carcinoma	External (e.g: paralateronasal approach)

Radiotherapy is a contentious treatment that has been generally considered for inverted papillomas in 3 situations: associated carcinoma, impossibility of surgery, and multiple recurrent lesions [27]. However, studies by Snyder and Prezin have demonstrated that radiation therapy is ineffective, and the potential for induction of malignancy and osteoradionecrosis has been cited as a contraindication to its use [28].

Given the risk of metachronous squamous carcinoma and recurrence, which may be late, IP follow-up duration and modalities are not codified, many authors recommend at least 6 years, while others suggest lifelong monitoring. Clinical examination and periodic flexible endoscopy are part of the follow-up. Biopsies can be performed if there is any doubt about a recurrence. Certain researchers advise MRIs every 4 months during the first postoperative year, then every 6 months for 4 years [29].

## Conclusion

Inverted papilloma is a benign nasal tumor well known for its invasiveness, tendency to recur and association with malignant tumors. Its diagnosis is difficult due to its low prevalence and the nonspecific symptomatology it shares with various pathologies [13]. Surgery must be performed with care, with prior radiological examination of the tumor extension to select the optimal procedure, which will remove the tumor entirely and reduce the risk of recurrence [26].

## CRediT authorship contribution statement

**Mohammed Amine Rabhi:** is the first author, study concept or design, data collection, data analysis or interpretation, writing the paper. **Achraf Amine Sbai:** study concept or design, data collection, data analysis or interpretation, writing the paper. **Drissia Benfadil:** participated in the writing, supervised, and revised the manuscript. **Adil Abdenbi Tsen:** participated in the writing, supervised, and revised the manuscript. **Azzedine Lachkar:** supervised and revised the final manuscript. **Fahd Elayoubi:** supervised and revised the final manuscript.

## Patient consent

Written informed consent was obtained from the patient for publication of this case report and accompanying images. A copy of the written consent is available for review by the Editor-in-Chief of this journal on request.
